# A Case of Hydrophobia

**Published:** 1786

**Authors:** R. Hamilton

**Affiliations:** the Royal College of Physicians in London, and Member of the Medical, Physical, and other Literary Societies in London and Edinburgh


XX. *A Case of Hydrophobia.* Vide *Remarks on the Means of obviating the fatal Effects of the Bite of a mad Dog, or other rabid Animal: with Observations on the Method of Cure when Hydrophobia occurs; and the Opinion relative to worming of Dogs refuted. Illustrated by Examples.* By R. Hamilton, *M. D. of the Royal*

*Royal College of Phyſicians in London, and Member of the Medical, Phyſical, and other Literary Societies in London and Edinburgh.* 8vo. Ipſwich, 1785.

THE ſubject of this caſe, the particulars of which are given in a Letter to Dr. Hamilton, from Mr. Tuſon, ſurgeon at Boxford, in Suffolk, was a young gentleman, about fifteen years old, who was on a viſit at the houſe of a friend in Jermyn Street, London, on the 6th of December, 1784, when a ſtray dog came into the room. The Lady of the houſe taking notice of its being very thin, ordered a plate of meat to be ſet before it, which the dog ate. Of this dog the young gentleman took particular notice, and while he was ſtooping down to examine it, the animal turned from its meat, and bit him on the right ſide of the lower lip. He was immediately ſent in a coach to the houſe of Mr. John Hunter, who ſaw him within a few hours after the bite, and who loſt no time in applying a cauſtic to every part of the wound. Dr. Turton was alſo conſulted, and under the direction of theſe gentlemen, the Ormſkirk and Tonquin remedies were adminiſtered, niftered, and mercurial ointment was rubbed into his legs twice a day.

On the 24th of December he arrived at his father's feat in the county of Suffolk, and the day following, when Mr. Tufon firft faw him, the wound in his lip was healed, and he was in perfect health. He continued, however, to take the Tonquin medicine, and to perfevere in the ufe of mercurial frictions, but without any appearance of illnefs till January the 11th, 1785, when he complained greatly of a pain in his right ear, and foon after was attacked with head ach and ficknefs. Thefe fymptoms were fucceeded by a very reftlefs night, and the next morning, when Mr. Tufon faw him, he found him labouring under a confirmed hydrophobia. The remainder of the hiftory we fhall give in Mr. Tufon's own words: "I called," fays he, "for fomething for him to drink: as foon "as it was offered to him he was convulfed, "particularly about the throat, and drank it "with much agitation; this confirmed me in "my opinion: he complained exceedingly "of a pain in his head, and great thirft; "his pulfe was very quick, full, and hard, "and he lay toffing about in the bed. I "afked him, if he felt any uneafinefs in his

"lip ? He told me he felt pricking pains about "the part the evening before, and at that time "he felt a forenefs on touching it. I defired "that every affiftance that could might be pro- "cured. Two meffengers were difpatched for "two phyficians. In the interim, I gave him "three dofes of the above mufk medicines "every hour, and propofed bleeding him; but "as farther affiftance had been fent for, I de- "ferred it till they came: they agreed in my "opinion. I took about ten ounces of blood "from the arm; they wifhed to fee him take "fome liquid; he took it in his hand, put it "haftily to his mouth, and upon deglutition, "all the mufcles concerned in that action "feemed very much convulfed. His tongue "appeared clean, not dry. They ordered "him—R. Cinnab. nat. & fact. aa gr. viij— "Mofch. gr. x. — Opii. gr. j. — Conferv. "Cynofbat. gr. ij. Syr. q. f. ft pil. ij.—Statim. "fumend. & tert. quaq; hor. repetend. fine "Opio. Thefe he took regularly; likewife "an injection with a pint of gruel, and two "ounces of oil, thrown up for a clyfter. A "cloth wetted with oil was applied to his "throat: his feet were bathed with flannels "dipped in hot water for a confiderable time

"toge-

" together, and four fcruples of the ftrong " mercurial ointment were rubbed in twice a " day, as they wifhed to promote a ptyalifm. " He paffed moft of the day in a chair, and " now and then walked about the room. His " eyes appeared very wild and red. He had " a fmall ftool from the ufe of the injection. " He made very little urine; his blood ap- " peared a little inflamed, and fomewhat fizy. " In the courfe of the day he frequently took " bread moiftened in tea or gruel. He " went to bed in the evening early, and got " two or three hours fleep (I fuppofe from " the effect of the grain of opium) after which " he appeared very reftlefs, and convulfed; to- " wards the morning he was exceedingly fo, " and not able to lie ftill a moment, conftantly " calling for fomething to drink, and com- " plaining of great thirft. He then fwallowed " with great anxiety and perturbation, and ap- " peared in the utmoft diftrefs.

" On the morning of the 13th (Thurfday) he " rofe about ten o'clock in the ftate above menti- " oned; at twelve his phyficians faw him again: " his pulfe was then about one hundred and thir- " ty. They then ordered him — R. Mofch. gr. " xij.—Mercur. Emet. flav. gr. ij.—Opii. gr. j.

" Mucilag. G. Arab. q. ſ. ut ft. Pil. No. ij

" tertia quaque horâ repetendæ cum vel ſine

" opio prout res poſtulare videatur,—and con-

" tinued the uſe of the ointment.—They had

" not left him an hour, before he was taken

" with frequent vomitings, retchings, and

" conſtant ſpitting of a viſcid phlegm. This

" came on before he took the Turbith pills.

" He took one doſe with opium as ſoon as it

" came, which was about two o'clock. He

" was very ſenſible and pertinent in his anſwers

" and converſation till now, when a delirium

" came on, with ſuch fears and horrors as are

" ſcarcely to be deſcribed—rubbing his throat,

" and walking up and down the room in great

" agony, but without offering violence to any

" one in it. He continued in this diſtreſ-

" ſed ſtate till about ſix in the evening, when

" he was ſtanding up, and leaning on the houſe-

" keeper; and then nature, from his inceſſant

" talking and raving, being exhauſted, he

" dropped down in a kind of fit. He was then

" laid on a bed quite ſenſeleſs, and ſpeechleſs,

" groaning, foaming at the mouth, now and

" then vomiting a dark brown choler, and ap-

" peared as if ſtrangled. He expired about

" half

" half paſt eleven at night. His lip after " death did not appear altered."

To Mr. Tuſon's account of this unfortunate caſe, Dr. Hamilton has added the following letter, written to him on the occaſion by Mr. John Hunter.

" Sir,

" I received the favour of yours. I am al- " ways extremely happy when I can give any " uſeful information; but all the information " I can give you relative to the Hydropho- " bia, is rather negative good than poſitive. " All the means recommended were uſed " in Maſter R.'s caſe. I ſaw him only a few " hours after the bite. The lip was torn a " good deal. The teeth had gone through " and through, and had torn out a piece. I " immediately applied the cauſtic to every ſur- " face that I conceived had been made by the " dog's teeth; and when thoſe ſloughs came " away, I went over the ſame field a ſecond " time; but, from the termination of the " whole, I am inclinable to believe, that I did " not touch every part where the teeth had " been. He took the Ormſkirk medicine by " the directions of Mr. Barry, who ſells it,

" therefore we muſt ſuppoſe it was properly

" given. He alſo took the Tonquin medicine,

" viz. muſk, cinnabar, &c. and rubbed in

" mercurial ointment till his mouth was ſore.

" My whole dependence was on the cauſtic,

" but, I did not object to the others being

" given. I wiſh I could ſay more on the ſub-

" ject in general. We ſeem to be as much

" at a loſs how to treat it as they were a thou-

" ſand years ago. I have not yet heard of the

" particulars of Maſter R.'s attack and ſymp-

" toms. I want very much to learn them.

" To aſcertain a mode of cure will be very

" difficult. For a few caſes not having the

" ſymptoms, under any courſe, prove but lit-

" tle. I know where there were twenty-one

" people bit by one dog; nothing was done

" for any of them, and only one was taken

" ill. If they had all taken medicines, then

" it would have been ſaid, that they only loſt

" one out of twenty-one.

" I am, dear Sir,

" Your moſt obedient ſervant,

" JOHN HUNTER"

In the preceding letter we find Mr. Hunter very candidly acknowledging, that, notwithſtanding all his care in this caſe, ſome part of the ſurface, which had been in contact with the dog's teeth, might perhaps eſcape the action of the cauſtic*. This remark, from ſo able a practitioner, would induce us, under ſimilar circumſtances, to give the preference (where it is practicable) to the exciſion of the part bitten, previouſly to the application of a cauſtic. This mode of prevention by exciſion, certainly derives as much weight as a ſingle caſe can give it, from a curious fact related by Dr. Hamilton, on the authority of Mr. Newſon, ſurgeon at Woodbridge, in Suffolk, in the work to which we are indebted for the preceding hiſtory. The caſe we allude to is that of a ſervant maid who was bit by a dog in the fleſhy and naked part of her arm. The lacera-

* From the ſame dog that bit maſter R. a poor French woman received a bite on one of her hands the ſame day. The ſore had cauſtic applied to it more than once, but not till ſeveral days after the bite. On Tueſday March 15th (the ninety-ninth day after the accident) ſhe began to complain of pain in the cicatrix; on the 19th ſhe had all the ſymptoms of hydrophobia, and ſhe died on the 22d.

tion extended four inches in circumference; and within two hours after the accident, the whole of the part bitten was removed by excision, and to the extent of at least half an inch, both in depth and width, farther than the dog's teeth had penetrated. The wound was afterwards dressed with escharotics, and was kept open six or seven weeks, after which it cicatrized without any difficulty, and the patient remained well. The dog that bit this young woman, likewise bit two other dogs, and both of these, we are told, were secured, and in the course of eight or nine days discovered the strongest signs of madness.

